# Epstein-Barr virus (EBV)-encoded small RNAs (EBERs) associated with poor prognosis of head and neck carcinomas

**DOI:** 10.18632/oncotarget.16033

**Published:** 2017-03-09

**Authors:** Turunen Aaro, Rautava Jaana, Grénman Reidar, Syrjänen Kari, Syrjänen Stina

**Affiliations:** ^1^ Department of Oral Pathology, Institute of Dentistry, University of Turku, Turku, Finland; ^2^ Department of Pathology, Turku University Central Hospital, Turku, Finland; ^3^ Department of Otorhinolaryngology - Head and Neck Surgery, Turku University Hospital, Turku, Finland; ^4^ Department of Clinical Research, Biohit HealthCare Oyj, Helsinki, Finland

**Keywords:** Epstein-Barr virus, EBV, head and neck cancer, HPV, HSV-1

## Abstract

**BACKGROUND:**

Epstein-Barr virus (EBV) is the main cause of nasopharyngeal carcinoma (NPC), also found in other head and neck carcinomas (HNSCCs) where its role remains controversial.

**RESULTS:**

EBV was found in 80% and 21% of the samples with PCR and ISH (in cancer cells), respectively. Eight of ISH-positive samples were not NPCs. EBER-RNA detection in carcinoma cells was associated with worse prognosis, whether or not NPCs were included. HPV/EBV and HSV/HPV coinfections associated with a shorter survival. LMP-1 expression, positive in 51% of samples did not correlate with the disease outcome.

**MATERIALS AND METHODS:**

We analyzed EBV in 73 HNSCC samples with a known HPV and HSV-1 status, using in situ hybridization (ISH) and immunohistochemistry (IHC) for EBV-early transcripts (EBER) and LMP-1 protein, respectively. EBV-DNA was detected with a Luminex-based method. The results were correlated with HPV-status and disease outcome.

**CONCLUSIONS:**

EBV is transcriptionally active in NPC cells but also in a subgroup of other HNSCCs.

## INTRODUCTION

Head and neck cancers (HNSCCs) are common cancers worldwide with over half a million annual new cases and over 370000 deaths [1]. An increasing subset of these cancers is known to be caused by human papillomaviruses (HPV)[2]. Epstein-Barr virus (EBV) is the main causative agent of nasopharyngeal cancers (NPC) [3, 4]. Recently, HPV has also been implicated as a potential inducer of these tumors [5]. Although HPV presence has been suggested as a marker of a more favorable prognosis in HNSCC patients [2] this may not always be the case [5], advocating further studies to elucidate this controversy.

Although detected in HNSCCs as well, EBV has not been confirmed in their pathogenesis despite increasing research interest [6–11]. Problems in confirming the role of EBV in HNSCC are ascribed to the fact that EBV infects B-lymphocytes latently in the majority of adults [12]. Thus, most HNSCC samples are testing EBV-positive with PCR-based methods because of its presence in the tumor-infiltrating B-lymphocytes. EBV is also known to replicate in epithelial cells of the oropharynx [4]. In immunocompromised subjects, productive EBV infection in epithelium presents as benign hairy leukoplakia lesions in the tongue [13].

EBV contributes to NPC pathogenesis mainly by its LMP-1, LMP-2A, EBNA1 and BARF1 oncogenes [3, 4]. EBERs are EBV-encoded small noncoding RNAs expressed in almost all EBV-infected cells and used as markers of an active EBV infection. EBERs disable innate immunity-related signaling in infected- and nearby cells [14]. LMPs are membrane proteins that activate signaling pathways beneficial to EBV persistence, whereas EBNAs regulate gene expression [4]. LMP-1 causes NF-κB pathway activation that leads to up-regulation of Bcl-2 oncogene and hTERT, chromosomal instability and compromised DNA repair, contributing to EBV-mediated malignant transformation [3, 15–17]. LMP-2A causes PI3K-mediated AKT activation leading to p53 degradation and c-myc oncogene over-expression, impairing the ability of the cell to undergo apoptosis [4, 18, 19]. EBNAs and LMP1 are implicated in cell responsiveness to radiotherapy [20]. Because HPV/EBV coinfection has been shown to enhance the invasiveness of HNSCC cells [10], our aims were to study the prevalence and localization of EBV in HNSCC with a known HPV status in order to reveal its possible effect on disease outcome. We hypothesized that because EBV affects NPC carcinogenesis it is likely that, if present in non-nasopharyngeal carcinoma cells, EBV has an effect on disease outcome in these tumors as well. The concomitant presence of HPV could theoretically also further increase the aggressiveness of these carcinomas via the expression of its well-known oncogenes.

## RESULTS

### Detection of EBV

**Figure 1:**
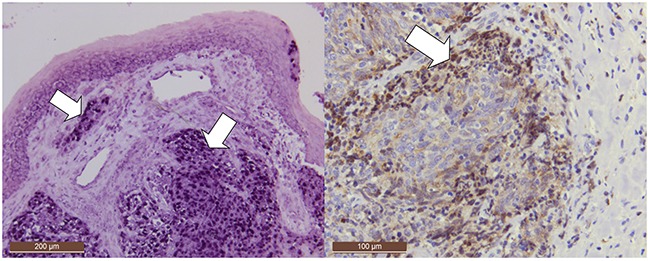
Positive controls: EBER positivity in a nasopharyngeal carcinoma (NPC) with EBER-ISH (deeper purple, mostly nuclear staining, arrows) EBER is intensively expressed in all NPC cells. Note that EBER is also expressed in localized area of normal surface epithelium. Additionally, LMP-1 was detected by immunohistochemistry in the cancer cells of invasion front in addition to occasional lymphocytes in that region (dark brown, 50x and 100x magnifications, respectively).

Table 1 summarizes the EBER RNA expression according to the localization of HNSCC, the presence of HPV and/or HSV-1. All NPCs except one showed intense, primarily nuclear expression of EBER RNA (Figure 1). The only case negative for EBER expression was also EBV PCR-negative, i.e., truly EBV-negative.

**Table 1:** EBER RNA expression according to the anatomic localization of HNSCC and presence of EBV DNA (PCR), multiple- or single types of HPV (HR- or LR type) or HSV-1 by PCR. Percentages given in parentheses present the positivity calculated from the total number of samples. The results of LMP-1 immunostaining are given as the number of cases with positive staining present in over 50% of the cancer cells

Site/n	EBER-ISH + carcinoma	EBER-ISH + lymphocytes	EBV PCR +	LMP-1 IHC +	HPV multiple	HPV single	HPV negative	HSV-1 PCR +
**Lip n=5**								
EBV +++	-	-	-	-	-	-	-	-
EBV +	-	-	-	-	-	-	-	-
EBV -	5 (100%)	2 (40%)	4 (80%)	1 (20%)	3 (60%)	2 (40%)	-	-
**Oral n=24**								
EBV +++	-	-	-	-	-	-	-	-
EBV +	2 (8%)	2 (8%)	2 (8%)	1 (4%)	-	1 (4%)	1 (4%)	-
EBV -	22 (92%)	8 (33%)	14 (58%)	4 (17%)	7 (29%)	8 (33%)	8 (33%)	1 (4%)
**Oropharynx n=19**								
EBV +++	-	-	-	-	-	-	-	-
EBV +	2 (11%)	2 (11%)	1 (5%)	1 (5%)	-	-	2 (11%)	-
EBV -	17 (89%)	5 (26%)	11 (58%)	3 (15%)	2 (11%)	7 (37%)	8 (42%)	2 (11%)
**Hypopharynx n=9**								
EBV +++	-	-	-	-	-	-	-	-
EBV +	1 (11%)	1 (11%)	-	-	-	1 (11%)	-	-
EBV -	8 (89%)	3 (33%)	7 (78%)	1 (11%)	3 (33%)	1 (11%)	4 (44%)	-
**Nasopharynx n=8**								
EBV +++	6 (75%)	2 (25%)	6 (75%)	6 (75%)	1 (13%)	1 (13%)	4 (50%)	-
EBV +	1 (13%)	1 (13%)	1 (13%)	-	-	1 (13%)	-	-
EBV -	1 (13%)	1 (13%)	-	1 (13%)	-	-	1 (13%)	-
**Larynx n=8**								
EBV +++	1 (13%)	1 (13%)	1 (13%)	1 (13%)	1 (13%)	-	1 (13%)	-
EBV +	1 (13%)	1 (13%)	N/A	-	-	1 (13%)	-	-
EBV -	6 (76%)	-	4 (50%)	-	-	2 (25%)	3 (38%)	-

N/A = Data not available

**Figure 2:**
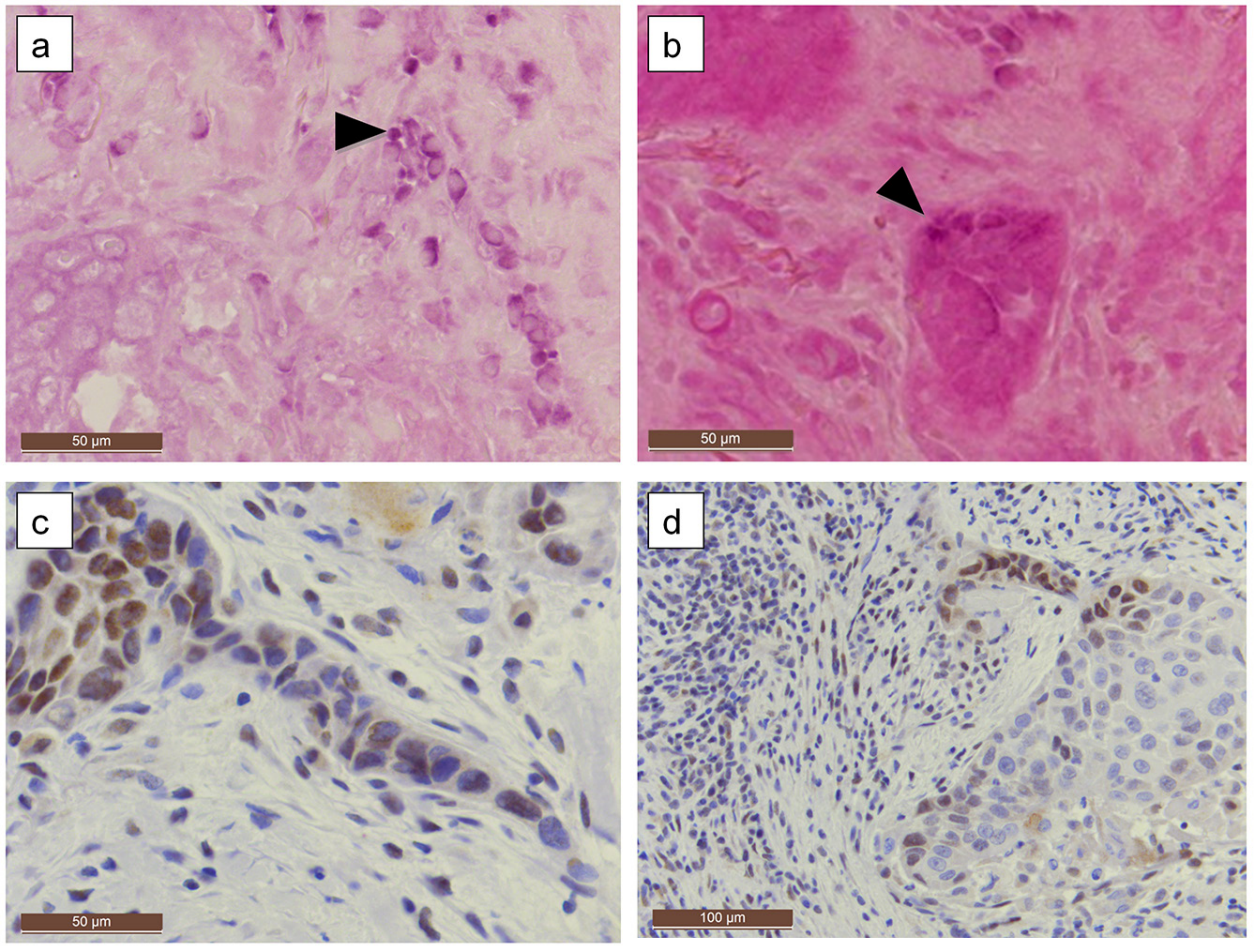
HPV genotype 6 positive tongue SCC EBER RNA expression was localized in carcinoma cell tract **(a)** in a patchy staining pattern and in infiltrating nests **(b)** (purple staining, arrowheads). LMP-1 was expressed simultaneously along the invasion front, especially in a thin invading tract (**c, d** immunohistochemistry, brown staining). (**a-c** Original magnification 200x, d:100x).

EBV transcripts were localized in cancer cells, lymphocytes, or in both in 21% (15/73), 41% (30/73), and 15% (11/73) of the HNSCC samples, respectively. EBER expression in cancer cells was intense in seven samples, of which six were NPC and one was laryngeal carcinoma. EBV transcripts were also found in a sub-population of cancer cells in eight cases, of which one was NPC, two tonsillar-, one hypopharyngeal-, two laryngeal- and two tongue carcinomas (Figure 2). Of the EBER positive samples, 73% were also EBV DNA positive with PCR, while 74% (23/31) of all cancers staining EBER-ISH positive in lymphocytes or cancer cells were simultaneously EBV-PCR positive. Of the PCR-positive cases, 44% (23/52) were also ISH-positive. NPCs excluding, 7% (4/57) of HNSCCs tested EBV positive with both ISH (in cancer cells) and PCR. These comprised two oral-, one laryngeal- and one oropharyngeal carcinoma. In the non-NPC cells, EBER expression was often cytoplasmic in addition to nuclear expression. EBER was expressed predominantly in carcinoma cells in the invasion front (Figure 2). NPCs and laryngeal carcinomas were most likely to express EBERs, followed by oro- and hypopharyngeal carcinomas (p=0.0001).

### LMP-1 expression in HNSCC samples

**Figure 3:**
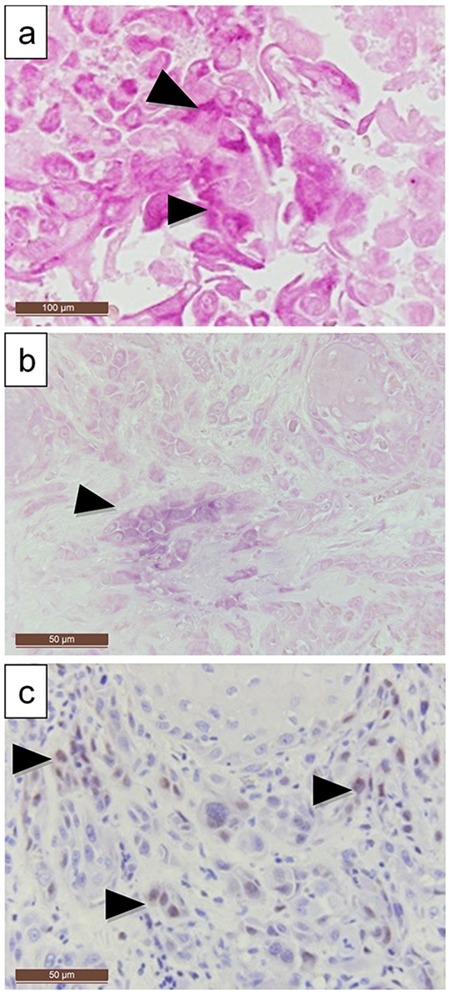
LMP-1 expression compared to EBER expression in a larynx carcinoma **(a, b)** EBER expression is localized in the poorly differentiated infiltrating tumor islands, **(c)** LMP1 can be detected simultaneously with immunohistochemistry **(a)** 200X and **(b)** and **(c)** 100X magnification).

Moderate to intensive LMP-1 immunostaining (Figure 1, Figure 2) was detected in 51% (32/62) of samples, whereas only 15% (9/62) of HNSCCs were LMP-1 negative (Table 2). LMP-1 positivity correlated with EBER RNA expression (p=0.031) and was mainly nucleo-cytoplasmic, localized also in cell membranes and mostly detected at the invasion front (in 48% of all tumors) alongside the positive lymphocytes (in 29% of all tumors) (Figure 2 and Figure 3).

**Table 2:** LMP-1 expression in carcinoma cells and lymphocytes of 62 HNSCC samples

	LMP1 in carcinoma cells			LMP1 in lymphocytes	
Staining	n	Percent	Staining	n	Percent
Negative	9	15%	Negative	36	58%
1-25%	21	34%	5%	10	16%
26-50%	14	23%	10%	9	15%
51-75%	10	16%	20%	4	6%
76-100%	8	13%	30%	3	5%
Total	62	100%		62	100%
	**LMP1 staining intensity**			**Localization of positive cancer cells**	
	**n**	**Percent**		**n**	**Percent**
Negative	9	15%	Negative	9	15%
Faint	9	15%	Invasion	30	48%
Moderate	23	37%	Patchy	11	18%
Strong	21	34%	Diffuse	12	19%
Total	62	100%		62	100%
	**Localization of positive lymphocytes**				
	n	Percent			
Negative	36	58%			
Invasion	18	29%			
Patchy	8	13%			
	62	100%			

All EBER-ISH-positive cancers tested positive for LMP-1 protein. Only three of the eight samples with >75% LMP-1 positivity showed EBER expression in cancer cells although all were PCR positive. Over 75% of the carcinomas with EBV PCR positivity and HPV coinfection were also positive for LMP-1 (p=0.005). Interestingly, areas with dysplastic and/or histologically normal basal cells were often LMP-1 positive in the immediate vicinity of carcinomas.

### The prevalence of coinfections

HPV, HSV-1, HSV-2 and CMV status of the HNSCCs has been reported previously by our group [21]. Here we compared their presence with the EBV data. HPV DNA of any type was found in 47% (7/15) and 60% (35/58) of the EBER RNA cancer cell positive and -negative HNSCC samples (difference p=0.602). Two EBV positive laryngeal carcinomas and three NPCs tested also HR-HPV-positive (HPV-16, 31/56 and 11/16, 16, 16, respectively) (Table 1). One tongue carcinoma with EBER RNA was HPV-6-positive. There were two HNSCCs having coinfection with EBV, HSV-1 and HPV; one oral carcinoma with EBV, HSV-1 and HPV-18 and another one with EBV, HSV-1 and HPV-6, 16 and 66. HSV-2 or CMV was not detected in any of the HNSCCs [19].

### Survival

NPCs were treated more often with chemoradiotherapy (CRT) than surgery only (p=0.024). Otherwise, there were no differences in treatment regimes. Overall disease-specific survival (DSS) was 43% for patients treated with curative intent. EBV detection with PCR was significantly associated with tumor size (TNM classification, T-class) (p=0.015), larger T4 tumors showing less EBV-positivity; 42% of the tumors in the PCR-negative group were T4 as compared to 6% in the PCR-positive group. PCR positivity to EBV was associated with a lower tumor grade in OSCCs (p=0.033) but not in other HNSCCs. No such association was found in ISH positive patients (p=0.906).

### EBER expression

**Figure 4:**
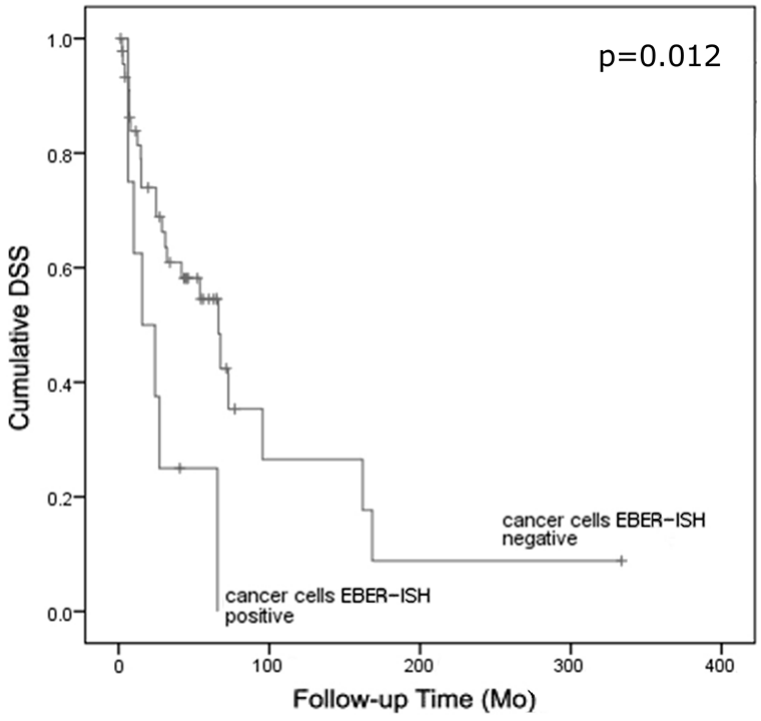
Cumulative disease-specific survival stratified by EBER mRNA expression in cancer cells of patients treated with curative intention

Patients with EBER RNA expression in the carcinoma cells (p=0.012) had shorter survival (30 months) than the EBER-negative patients (88 months) (Figure 4). This was independent of the EBER expression in lymphocytes (p=0.039). Excluding NPC cases from the analysis did not change these results (p=0.012).

### Coinfections

**Figure 5:**
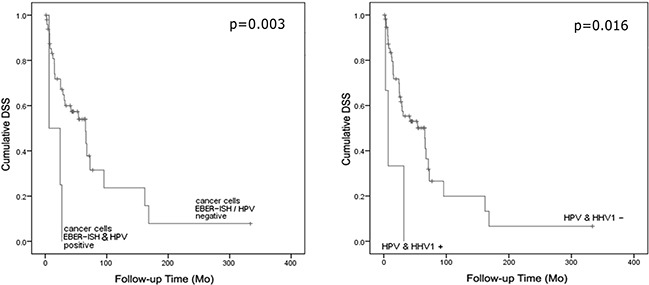
Cumulative disease-specific survival as stratified by either (a) EBER mRNA expression in cancer cells and HPV coinfection (positive/all cases n=4/54, p=0.003) or (b) HPV/HSV-1 coinfection (positive/all cases n=3/60, p=0.016)

Excluding NPCs; four EBER-positive carcinomas (2 laryngeal, 1 oral and 1 hypopharyngeal SCC) co-infected with HPV (types 31/56, 16, 6 and 16, respectively) had a mean survival time of 16 months as compared to the patients without coinfection (84 months average, p=0.003, Figure 5A). A similarly poor survival was apparent in three HSV-1/HPV–co-infected patients (two tonsillar- and one palatal carcinoma, testing positive for HPV-16, 16 and 18, respectively) (p=0.016, Figure 5B). Two of them were also EBV DNA positive.

The survival of patients with EBV and HPV- coinfected HNSCC was related to the presence/transcription of EBV as detected either with PCR (EBV DNA) or ISH (EBER mRNA) (p=0.008). Survival time was shortest in patients where EBER was present as a coinfection with LR-HPV (9 months) followed by the coinfection with HR-HPV (19 months). The survival was longest for patients with EBV DNA and LR-HPV positive cancers (90 months), followed by EBV and HPV negative cancers (85 months) and EBV DNA and HR-HPV positive cancers (54 months).

### Immunohistochemistry

Expression of LMP-1 did not correlate with the disease outcome or stage. LMP-1 was mostly expressed in areas with EBER positivity as well, although LMP-1 staining was more prevalent.

## DISCUSSION

We demonstrated the presence of EBER transcripts in the tumor cells of HNSCCs other than NPC. We also found that EBV affects the disease outcome. Although the presence of HPV/EBV coinfections on DNA level is well known, the present study is among the first [10,11] where the topographic expression of EBV (i.e., cancer cells or lymphocytes) in HNSCC was analyzed according to the coinfections with other viruses; HPV, CMV and HSV, and their relevance to disease outcomes in head and neck region. Also, to our knowledge, this is the first study to show an association between HPV/EBV coinfections and HNSCC prognosis in an European country.

Recently, Jiang et al. [10] found HPV/EBV coinfections in up to 25% of tonsillar carcinomas. Our results are in alignment with these findings. However, 4/16 of their tonsillar carcinomas tested HPV- and EBV-ISH positive, whereas none of our cases did. This could be due to differences in geographic variation or patient characteristics. The authors [10] postulated that EBV infection might be distributed anatomically more widely than formerly appreciated. Our results support this hypothesis. Moreover, they described carcinomas that were EBER-1 positive in only certain areas of the tumor tissue, much like our findings of partially positive carcinomas. Interestingly, the increased invasiveness of HPV/EBV co-infected epithelial cells due to LMP-2- and E6/E7-oncogene coexpression [10] might perhaps explain the ominous disease outcome for such patients as found in our study.

The invasive fronts in our cancer samples were often EBER and LMP-1 positive, particularly within budding carcinoma cell clusters which are known to be important prognostic markers [23]. Also, tumor infiltrating lymphocytes seemed to display positivity at the invading front. It can be hypothesized that the presence of EBV in the invasion front might augment the invasive potential of HPV-positive cancer cells, resulting in poor survival. Controversially, recent findings link HPV infections with an increased risk of distant metastases in oral cancer patients [24]. Also, emerging evidence suggests LR-HPVs as predictors of poor survival [25]. Thus, the presence of other viruses, particularly EBV, should be further studied in HPV-positive carcinomas.

In the comprehensive study by Deng and coworkers [11], 69.9% and 6.2% of HNSCCs were EBV-PCR and ISH–positive, respectively. Also, HPV-positivity in OPCs was associated with survival differences, whereas EBER-expression was not. They identified only one case of non-NPC with EBER expression using the same method as us. Approximately 22% of their patients had HPV/EBV coinfection detected with PCR. However, only 2 (1%), NPCs were both HPV DNA and EBER-positive. Our results showed that 8/15 EBER positive carcinomas were not NPCs, and 4 HNSCCs (∼6%) had similar HPV/EBV coinfections in our study. These differences might reflect differences in viral prevalence among the patients from Okinawa (Japan) and Finland [26].

Because EBV resides in B-lymphocytes that infiltrate tumor sites, the types of the EBV infected cells in a tumor sample cannot be estimated in PCR-based studies. However, this fact has frequently been ignored in the literature. Accordingly, Nasher and coworkers found no correlation between oral carcinomas and EBV [27], despite the high EBV detection rate by PCR (73%). We also found a nearly similar EBV prevalence with PCR- but only 8% of them showed EBER expression. Another recent study reported the association between the grade of OSCC and the presence of EBV as detected by PCR only [9]. EBV was detected in 17% of the samples but its presence was not related to the tumor grade. Contradictory to that we found that EBV DNA positivity was associated with a lower grade of OSCC.

The presence of EBER transcripts in cancer cells was associated with a poor prognosis while EBV DNA positivity in general (with PCR) without specifying the origin of the infected cells whether cancer cells or lymphocytes was associated with less overall HNSCC deaths, smaller tumors and lower OSCC grade. This is plausible, since immune responses are known as signs of favorable prognosis, limiting cancer growth and possibly contributing to the increased quantity of EBV genomes in infected lymphocytes as detected with PCR [28]. As EBERs were expressed only in a subset of carcinoma cells, EBV could have spread from inflammatory cells, to the adjacent basal cells showing EBER- and LMP-1 positivity. In hairy leukoplakia it is known that EBV produced by lymphocytes in lamina propria can infect the basal cells of tongue epithelium.

Treatment resistance was observed in carcinomas with HPV/HSV-1 coinfections [21]. We recently showed that HSV-1 infection alters the radio-response of oral keratinocytes [29]. In fact, most of these patients underwent radiotherapy, emphasizing the importance of understanding the role of coinfections in HNSCC after radiotherapy. Only 11% of the HNSCCs in our series tested negative for all viruses, suggesting that viral infections in HNSCC are more common than expected. Lastly, EBV has recently been shown to replicate in differentiating epithelia [30], making the role of EBV in malignant transformation more intriguing.

Our study is not flawless in its design, however. Failure to analyze all cases in the original study [21] due to insufficient samples available and lack of comprehensive data on the use of tobacco and alcohol hamper the statistical analysis between these studies. Also, studies with a larger sample sizes are required in the future.

To conclude, EBV is present in a subset of HNSCC of non-nasopharyngeal origin. Using a highly sensitive ISH, EBV is detected in the cancer cells, whereas the majority of EBV detected by PCR originates from lymphocytes. Our study provides evidence that EBV/HPV coinfections might represent a sign of unfavorable prognosis in HNSCC patients.

## MATERIALS AND METHODS

### Samples of the HNSCC patients

Paraffin-embedded tumor (FFPE) samples from 73 patients (27 women (37%) and 46 men (63%) with HNSCC diagnosed and treated at the Department of Otorhinolaryngology, Turku University Hospital, during 1988-2009 were included [21]. Frozen tissue samples were available from 64 of these patients. The mean age of these patients was 63 years (Std. 13.9 years). The patients were followed up for an average of 39.1 (Std.48.3) months. From available patient data, 82% were smokers and 80% consumed alcohol (n=28 and n=15 respectively). 90% were treated with curative intention. Table 3 summarizes the treatment, HPV presence and staging of the patients’ tumors. The diagnoses of all tumors were confirmed by histopathological re-examination.

**Table 3:** HPV status, staging and treatment modes of the HNSCCs analyzed. Also, the TNM (tumor, neck metastasis, distant metastasis) classification is presented

High-risk/low-risk-HPV-positive (PCR) % (n)	
Lip (n=5)	80%/80% (4/4)
Oral (n=24)	52%/43% (12/10)
Oropharyngeal (n=19)	47%/11% (9/2)
Hypopharyngeal (n=9)	55%/22% (5/2)
Laryngeal (n=8)	50%/0% (4/0)
Nasopharyngeal (n=8)	38%/13% (3/1)
**TNM**	
T1	14 (19%)
T2	33 (46%)
T3	15 (21%)
T4	10 (14%)
N0	37 (54%)
N1	15 (22%)
N2	13 (19%)
N3	4 (6%)
M0	68 (99%)
M1	1 (1%)
**Stage**	
I	6 (10%)
II	18 (30%)
III	12 (20%)
IVA	19 (31%)
IVB	5 (8%)
IVC	1 (2%)
**Grade**	
1	20 (38%)
2	22 (42%)
3	10 (19%)
**Operative treatment**	45 (61%)
**Chemoradiotherapy**	14 (19%)
**Radiotherapy**	46 (63%)

### Detection of the viruses

The presence of HPV, HSV-1, HSV-2 and CMV (Cytomegalovirus) in these tumors has been reported earlier [21]. Analyzed genotypes include both low risk (LR)-HPV-types 6/11/42/43/44/70 and high risk (HR)-types 16/18/30/31/33/35/39/45/51/52/53/56/58/59/66/68/73/82. The presence of EBV DNA was analyzed with an in-house PCR and Luminex xMAP-based method [22] using DNA extracted from the frozen tissue samples [22]. EBV gene target sequences: Forward primer: 5’-GAC-TGT-GTG-CAG-CTT-TGA-CGA-T-3, Reverse primer: 5’-CAG-CCC-CTT-CCA-CCA-TAG-GT-3’. Luminex probe: 5’-GGA-AAC-CAG-GGA-GGC-AAA-TCT-A-3’ [22].

The presence of EBV RNA transcripts EBER1 and EBER2 was examined with the “Epstein-Barr Virus (EBER) PNA Probe/Fluorescein and PNA-ISH Detection Kit” (Dako, Glostrup, Denmark) according to the manufacturer's instructions on the FFPE cancer samples. The substrate was incubated for 60 minutes, followed by a counterstain with Eosin and mounted using Aquamount (Dako). EBER-expression was graded from negative (−) to slight (+), moderate or intensive (+++), where most cells express EBER-RNA

### Immunohistochemical staining for LMP-1

In total, 62 FFPE biopsy samples were available for a standard immunoperoxidase technique for the detection of LMP1 using monoclonal mouse anti-EBV LMP-1 antibody with a dilution of 1:50 Dako Real™ peroxidase/DAB+ rabbit/mouse kit and LMP-1 antibody (Dako) using the Dako TekMate -tissue stainer. Blocks unavailable for LMP1 staining were of insufficient size and adequate carcinoma tissue was not present for further analysis. For pretreatment, the slides were microwaved in a citrate buffer, pH 6.0 for 5 minutes, twice. Proteinase K pretreatment (10 minutes, Dako Real Proteinase-K ready-to-use kit) was performed using the TekMate. Positive control was a paraffin section from EBV-positive NPC. In negative control the primary antibody was omitted. The slides were analyzed separately by two authors (AT and SS). Percent immunopositivity in carcinoma cells and/or lymphocytes was reported as negative, 1-25%, 26-50%, 51-75% or 76-100% positivity. Disagreement between authors was resolved by consensus meeting. Localization of positive signals was classified as present in the i) invasion front (invasion), ii) scattered carcinoma cells (patchy) or iii) majority of the carcinoma cells (diffuse). In lymphocytes, the LMP-1 localization was classified as either i) adjacent to the invasive front, or ii) scattered around the carcinoma (patchy staining). The slides were photographed using a Leica DC500 camera with Leica application suite v4.2 (Leica Microsystems GmbH, Wetzlar, Germany).

### Statistical analysis

Analyzes were performed using SPSS (IBM SPSS Statistics for Windows with SPSS advanced statistical package, Ver.19.0.0.1, Armonk, NY:IBM Corp. 2010). The χ^2^-test was used for categorical variables utilizing the Fisher's exact test as needed. Odds ratios (OR) were calculated using the exact method with 95% CI. Mann-Whitney- or Kruskal-Wallis (for two or several samples, respectively) tests were used to detect differences in the means of continuous variables. Disease-specific survival (DSS) was analyzed using univariate survival (Kaplan-Meier) analysis, where the stratum-specific estimates were compared using the Mantel-Cox (log-rank) statistics. As in the previous study [21], only the patients treated curatively were included in the survival analyzes. Analyzes were performed two-sided, considering p-values of <0.05 significant.

## Abbreviations

EBV: Epstein-Barr virus small RNAs
EBER: Epstein-Barr virus (EBV)-encoded small RNA
HNSCC: head and neck carcinoma
NPC: nasopharyngeal carcinoma
HPV: Human papillomavirus
HSV-1: Herpes Simplex type 1
ISH: in situ hybridization
IHC: immunohistochemistry
LMP-1: latent membrane protein 1
EBNA1: Epstein-Barr virus nuclear antigen 1
BARF1: BamHI-A rightward frame 1
FFPE: Formalin fixed paraffin-embedded
CMV: Cytomegalovirus
CRT: chemoradiotherapy
DSS: disease-specific survival
